# Genomic landscape and subgroup stratification of thymic epithelial tumors: a systematic meta-analysis of next-generation sequencing data

**DOI:** 10.3389/fonc.2026.1781510

**Published:** 2026-02-26

**Authors:** Eleonora Pardini, Serena Barachini, Marina Montali, Irene Sofia Burzi, Gisella Sardo Infirri, Daniele Tagliafierro, Iacopo Petrini

**Affiliations:** 1 Department of Translational Research and New Technologies in Medicine and Surgery, University of Pisa, Pisa, Italy; 2 Department of Clinical and Experimental Medicine, University of Pisa, Pisa, Italy

**Keywords:** GTF2I, next generation sequencing, thymic carcinoma, thymic epithelial tumors, thymoma

## Abstract

**Introduction:**

Thymic epithelial tumors are rare cancers of the anterior mediastinum with heterogeneous clinical behaviors. Despite numerous attempts to characterize their mutational landscape, a comprehensive understanding of their genetic alterations remains limited due to small sample sizes.

**Methods:**

To address this gap, we conducted a systematic meta-analysis of somatic mutations reported in 729 patients across twenty studies, integrating single-variant data into a unified dataset.

**Results:**

This approach identified three molecular subgroups with distinct biological and clinical features. Tumors harboring GTF2I mutations were typically indolent, exhibited low mutational burden, and showed enrichment in pathways related to cell adhesion. Tumors with TP53 mutations displayed high mutational load, activation of receptor tyrosine kinase and mitogenic signaling, and corresponded to aggressive clinical behavior. Tumors lacking both GTF2I and TP53 mutations revealed intermediate pro les, characterized by alterations in epigenetic regulation and extracellular matrix organization. Mutational signature analysis indicated that age-related processes predominate in less aggressive tumors, while DNA repair de ciency characterizes those with TP53 mutations. Network and pathway analyses revealed convergent oncogenic hubs and distinct signaling dependencies across subgroups.

**Discussion:**

This large-scale integrative study provides a re ned map of the genetic landscape of thymic epithelial tumors, highlights biologically meaningful heterogeneity, and establishes a framework to guide future research and the development of targeted therapies.

## Introduction

Thymic epithelial tumors (TETs) are rare malignancies originating in the anterior mediastinum. According to the 2021 WHO classification, TETs are divided into thymomas and thymic carcinomas (TCs) (1). Thymomas resemble the structure of normal thymus and are further subclassified into A, AB, B1, B2, B3, micronodular, and metaplastic histotypes (1). Thymomas have heterogeneous clinical behavior: the micronodular, A, AB, and B1 types tend to be more indolent and commonly harbor GTF2I mutations but not copy-number aberrations (2). B2 and B3 thymomas have an invasive growth involving the mediastinal structures and the pleural cavity. B2 and B3 thymomas less frequently have GTF2I mutation but present a characteristic pattern of copy number aberrations (2). Myasthenia gravis is commonly associated with thymomas and rarely with carcinomas. TCs resemble features of carcinomas originating in other organs, most frequently squamous cell carcinomas (1). TCs are frankly aggressive tumors more commonly diagnosed in the advanced stages, present a 5-year survival of around 50% if resectable, and a median overall survival of around 24–30 months if metastatic (1). TCs frequently present mutations or deletions of CDKN2A, TP53, and CDKN2B (3, 4). Surgery is the mainstay of treatment for localized disease, whereas systemic drugs, including chemotherapy, anti-angiogenic drugs, and immunotherapy, are adopted for metastatic tumors (5). Although several attempts have been made to characterize these tumors at the molecular level, valuable therapeutic targets remain elusive. Due to the rarity of these tumors, sequencing analyses are limited to small series. Therefore, a comprehensive and systematic study of literature reporting next-generation sequencing results can help to decipher relevant pathways for TET subgroups. We performed a systematic meta-analysis of somatic mutations in TETs, identifying articles reporting mutation data at the single-variant level, and provided a comprehensive analysis of published results.

## Methods

This systematic review has been conducted in accordance with the Preferred Reporting Items for Systematic Reviews and Meta-Analyses (PRISMA) guidelines (6).

### Literature search strategy

**Figure 1:**
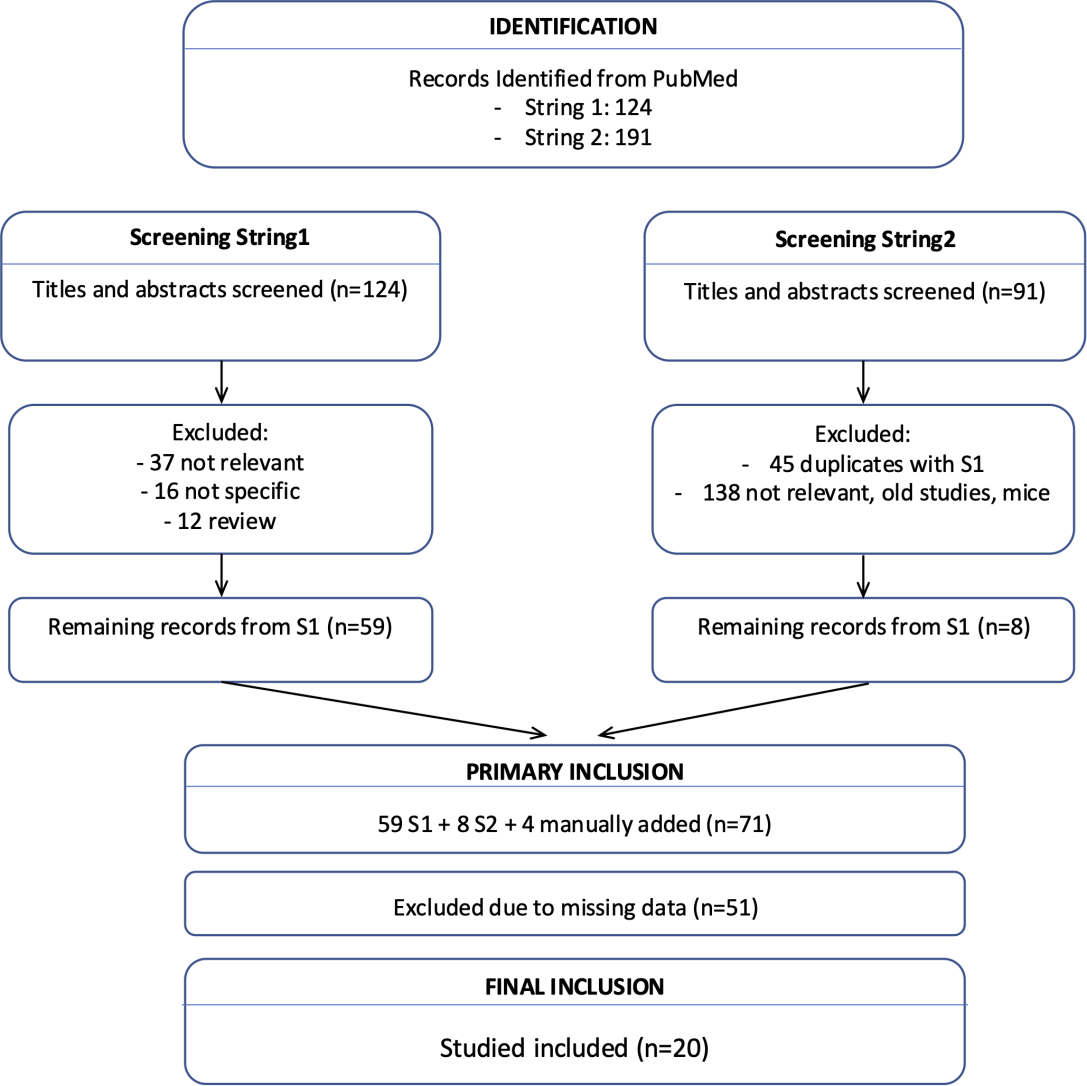
Flow diagram illustrating the study selection process for the meta-analysis, showing studies that met the inclusion criteria and those excluded in accordance with PRISMA guidelines.

A systematic literature search was performed in PubMed on December the 1^st^ 2025, using two predefined search strategies designed to maximize sensitivity while maintaining specificity (**Figure 1**). The first search strategy retrieved 124 records using the search key: (“thymic epithelial tumors” OR “thymoma” OR “thymic carcinoma”) AND (“next-generation sequencing” OR “NGS” OR “whole-genome sequencing” OR “exome sequencing” OR “targeted sequencing” OR “somatic”). The second search strategy yielded 191 records using the search key: (“thymoma” OR “thymic carcinoma”) AND (“genomic”).

Only articles published in English were considered eligible.

Articles were included if they: 1) Investigated the somatic mutational landscape of thymic epithelial tumors (TETs); 2) Employed next-generation sequencing-based genomic approaches (both whole genome, whole exome, and targeted sequencing); 3) Involved adult human populations; and 4) reported somatic mutations at the single variant level with coordinates and base substitution.

Articles were excluded if they: 1) Addressed diseases related to, but not specifically involving, thymic epithelial tumors; 2) Investigated molecular aspects of TETs without using next-generation sequencing technologies; 3) Involved non-human subjects, including animal models or cell lines; 4) Were conference abstracts without full-text availability; 5) Were narrative reviews or systematic reviews; 6) Reported mutations in an aggregate format without the possibility to define single variants.

### Article screening and data extraction

Two reviewers (EP and IP) independently screened all retrieved records by title and abstract. Studies were advanced to full-text review if deemed potentially eligible by at least one reviewer. The same reviewers independently assessed the full-text articles against the predefined inclusion and exclusion criteria. Data extraction was performed independently by both reviewers using a standardized form. It included the following variables: authors, article title, journal, year of publication, sample size, next-generation sequencing variant detection methodology, and human genome reference build. Both reviewers independently assessed methodological quality, and any discrepancies were resolved through discussion and consensus. Of the 315 records retrieved by the two search strategies, 20 studies met the inclusion criteria, addressed the research question, and provided sufficient data for meta-analysis. The included studies were published between 2013 and 2025. The study selection process is summarized in the PRISMA flow diagram. The number of included studies and publication years is reported in the results section and summarized in the PRISMA flow diagram.

### Creation of a unified VCF and variant annotation

From the selected 20 reports, somatic mutations at the single-variant level, including chromosome positions and nucleotide substitutions, were extracted, converted into hg19 positions by lift-over, and integrated to generate a unified Variant Call Format (VCF) file. The identity of each patient with each mutation was uniquely labeled, and patients’ data were stored under the same name in a file containing patient characteristics (**Supplementary Table 2**).

We used R version 4.5.2 (2025-10-31) and RStudio version 2025.09.2 + 418. Firstly, we annotated the mutation with SnpEff 5.4a (build 2025-11-25 12:22) (7) and then with Annovar (8). To ensure data integrity and avoid redundant entries in our meta-analysis, a unique genomic key was generated for each variant based on its sample ID, chromosomal coordinates, and specific allelic change. Technical duplicates were systematically removed using the distinct function in R, ensuring that each unique somatic event was represented only once per patient in the final integrated dataset (**Supplementary Table 3**). Descriptive statistics were used to summarize the clinicopathological characteristics and mutational frequencies of the cohort. For the comparison of continuous variables between two molecular subgroups, Welch’s t-test was applied to account for potential unequal variances. All statistical analyses were performed using the rstatix package (v0.7.2) (Available from: https://CRAN.R-project.org/package=rstatix) and the dplyr package (v1.1.4) (Available from: https://CRAN.R-project.org/package=dplyr).

The mutational landscape of the integrated cohort was analyzed and visualized using the maftools package (v2.26.0). Specifically, we used the package to generate Oncoplots for identifying the most frequently mutated genes and to calculate mutation frequencies within each histological subtype. To investigate the molecular relationships among key drivers, we performed a pairwise mutational co-occurrence and mutual exclusivity analysis, applying Fisher’s Exact Test to identify significant gene interactions. Additionally, mutational signature extraction was performed by fitting observed transitions and transversions against the COSMIC v3.2 database using the signature analysis module in the maftools environment (9). The mutational signature was stratified according to the molecular subgroups of TETs: 1) GTF2I-mutated samples harboring the L424H mutation; 2) TP53-mutated: Samples with non-synonymous mutations in TP53; 3) Double Negative (DN): Samples lacking mutations in both GTF2I and TP53. For each subgroup, the aggregate mutational profile was calculated by summing the counts across all 96 trinucleotide contexts. Similarity between the subgroup profiles and the COSMIC signatures was quantified using cosine similarity scores, ranging from 0 (no similarity) to 1 (identical profile).

### Estimation of mutation frequency and heterogeneity between studies of the most frequently mutated genes

To assess the frequency of mutations in the 10 most mutated genes in TETs, we use a meta-analytic workflow (10). We excluded from this analysis studies evaluating a single patient, since the frequency of mutation can not be estimated. We included only studies evaluating the mutation of the specific gene. For example, GTF2I was not included in most targeted resequencing panels. For the analysis, we used R with the “meta” package (11). To account for the expected clinical and biological diversity among the included studies, a Random Effects Model was primarily employed to calculate the pooled proportions and their 95% Confidence Intervals. The Inverse Variance method was used for weighting, and the DerSimonian-Laird estimator was applied to calculate the between-study variance. To stabilize the variance of proportions, especially for rare mutations (near 0%), the Freeman-Tukey double arcsine transformation was applied before pooling. Individual study Confidence Intervals were calculated using the Clopper-Pearson method. Statistical heterogeneity was assessed using Cochran’s Q test and quantified by the I2.

### Pathways analysis

To identify the core biological processes altered in our cohort, we conducted an oncogenic pathway analysis using the maftools package. Mutations were mapped to 10 canonical signaling pathways (including Notch, Wnt, Hippo, MYC, and RTK-RAS) based on the classification framework provided by Sanchez-Vega et al. (12).

Thereafter, we conducted a Reactome analysis of pathways affected by somatic mutation in TETs. Firstly, we selected driver mutations, manually curated to remove noise from passenger mutations, for pathway enrichment analyses. Statistical drivers were defined using the OncodriveCLUST algorithm (implemented in the maftools R package), which identifies genes under positive selection by clustering mutations by position relative to a background model. A minimum threshold of 3 mutations per gene and an adjusted p-value < 0.05 to control FDR were required for significance. To ensure a comprehensive genomic landscape, the statistical results were supplemented with a panel of clinically and biologically relevant genes in thymic malignancies, including GTF2I, SETD2, ASXL1, BCOR, TET2, KMT2D, ATM, BAP1, CYLD, NF1, SMAD4, and APC. This integrative approach enabled the inclusion of all the most frequently mutated genes in TETs that might otherwise escape clustering algorithms due to the non-random, distributed nature of loss-of-function mutations. Over-representation analysis was performed using the Reactome database via the ReactomePA and clusterProfiler packages. Gene Symbols were converted to Entrez IDs using the “org.Hs.eg.db” library. Statistical significance was determined using a hypergeometric test, with p-values adjusted for multiple testing via the Benjamini-Hochberg method (adjusted p< 0.05). To visualize the interconnectivity between genes and pathways, a Gene-Concept Network (Cnetplot) was generated, illustrating how specific hubs bridge multiple biological processes.

To characterize the biological landscape of the different molecular clusters (GTF2I-mutated, TP53-mutated, and Double Negative), we performed a functional enrichment analysis using the Reactome database. Mutation-associated gene sets for each cluster were mapped to biological pathways. Statistical significance was determined using a hypergeometric test. The BgRatio (the total number of genes in the genome associated with that pathway) was calculated. To account for multiple testing, p-values were adjusted using the Benjamini-Hochberg method to control the FDR. Pathways were considered significantly enriched if the adjusted p-value < 0.05.

Three primary metrics were used to evaluate pathway impact: 1) Rich Factor: The ratio of the number of BgRatio mapped to a pathway relative to the total number of genes in that pathway. 2) Fold Enrichment: Representing the magnitude of enrichment over the background. 3) z-Score: Calculated to indicate the overall activation or suppression trend of the pathway based on gene count and distribution.

To confirm the biological implications of the mutational landscape, we performed a Kyoto Encyclopedia of Genes and Genomes (KEGG) pathway enrichment analysis. This analysis was first conducted on the entire cohort to identify processes that were universally altered. Subsequently, to dissect the molecular heterogeneity of thymic epithelial tumors, the cohort was stratified into three distinct subgroups: GTF2I-mutated, TP53-mutated, and DNs lacking both mutations. Pathway enrichment was calculated using the overrepresentation of mutated genes within specific KEGG pathways. Statistical significance was determined using an enrichment score, and p-values were adjusted for multiple testing using the Benjamini-Hochberg method. This stratified approach allowed for the identification of subgroup-specific pathways.

## Results

Among the 20 reports selected (2, 13–31), we identified 729 patients for whom next-generation sequencing data have been reported. The median age was 57 years (range 17-86), and 49% were male (sex was reported for 309 females and 300 males). There were 166 TCs (23%) and 563 thymomas (77%); the patients’ clinical characteristics are reported in **Table 1**. Masaoka and Koga’s stage was the most commonly reported, and we combined stage IIA and IIB into a single stage II group because there were 92 tumors in stage IIA and 120 in stage IIB, but for 70 stage II cases, the distinction between stage IIA and IIB was not reported. Clinical characteristics of 304 patients without detected mutations have been reported and included in the analysis.

**Table 1:** Characteristics of the patients.

Median age	57	Range 17-86
Sex	49% male	F/M 309/300
WHO		
A	90	12%
AB	140	19%
B1	56	8%
B1/B2	4	1%
B2	93	13%
B2/B3	20	3%
B3	106	15%
MNT	20	3%
TMP	1	0%
TC	166	23%
TNEC	16	2%
Thymoma NOS	17	2%
Stage		
I	99	13%
II	282	39%
III	75	10%
IV-A	52	7%
IV-B	51	7%
UK	170	23%

M, male; F, female; MNT, micronodular; TMP, metaplastic thymoma; TC, thymic carcinoma; TNEC, thymic neuroendocrine carcinoma; NOS, not otherwise specified, UK, unknown.

**Figure 2:**
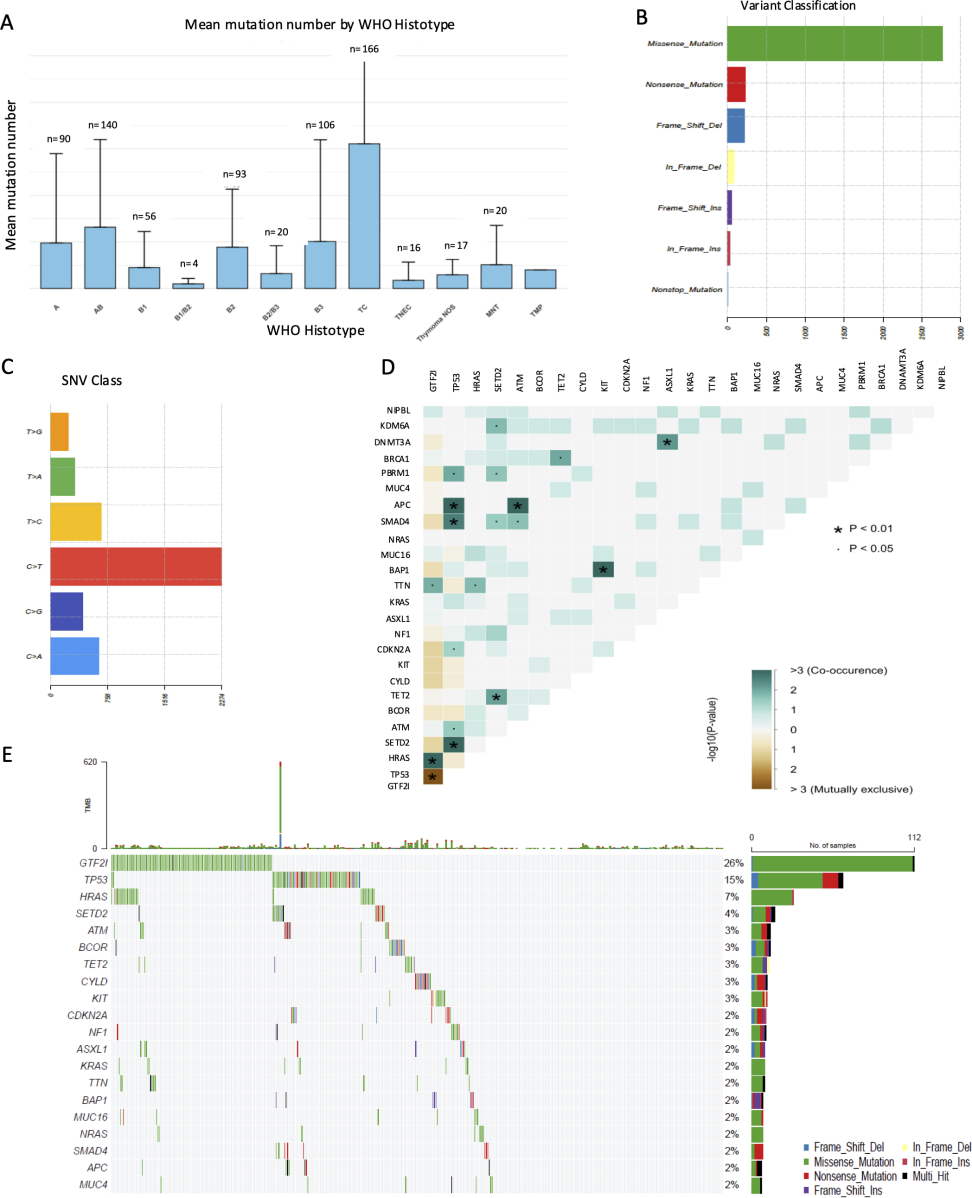
Characteristics of the observed mutations. **(A)** Bar chart showing the mean number of mutations (Y-axis) across the WHO histological subtype (X-axis). **(B)** Number of mutations categorized by exonic function: missense, nonsense, frameshift deletion, in-frame deletions, frameshift insertion, in-frame insertions, and non-stop mutations. Synonymous mutations were excluded. **(C)** Box plot illustrating the transition/transversion ratio. **(D)** Concordance table of co-occurring or mutually exclusive mutations. Significant co-occurrence is marked with ‘•’ for p < 0.05 and ‘*’ for p < 0.01. **(E)** Oncoplot displaying the most frequently mutated genes in TETs and mutation types, excluding synonymous mutations. This figure summarizes the results from 425 out of 729 samples with at least one mutation.

The total number of mutations was 5141. TCs had a trend for more mutations, with an average of 15.6 mutations/sample (standard deviation (SD) of 74.8), compared to thymomas, with an average of 4.7 (SD 8.5; Welch T-test p=0.0629; **Figure 2A**) The average number of mutation in stage I-II was 5.4 (SD 9.11) and 14.8 in stage III-IV (SD 72.1; Welch Test-T p=0.0848).

**Figure 3:**
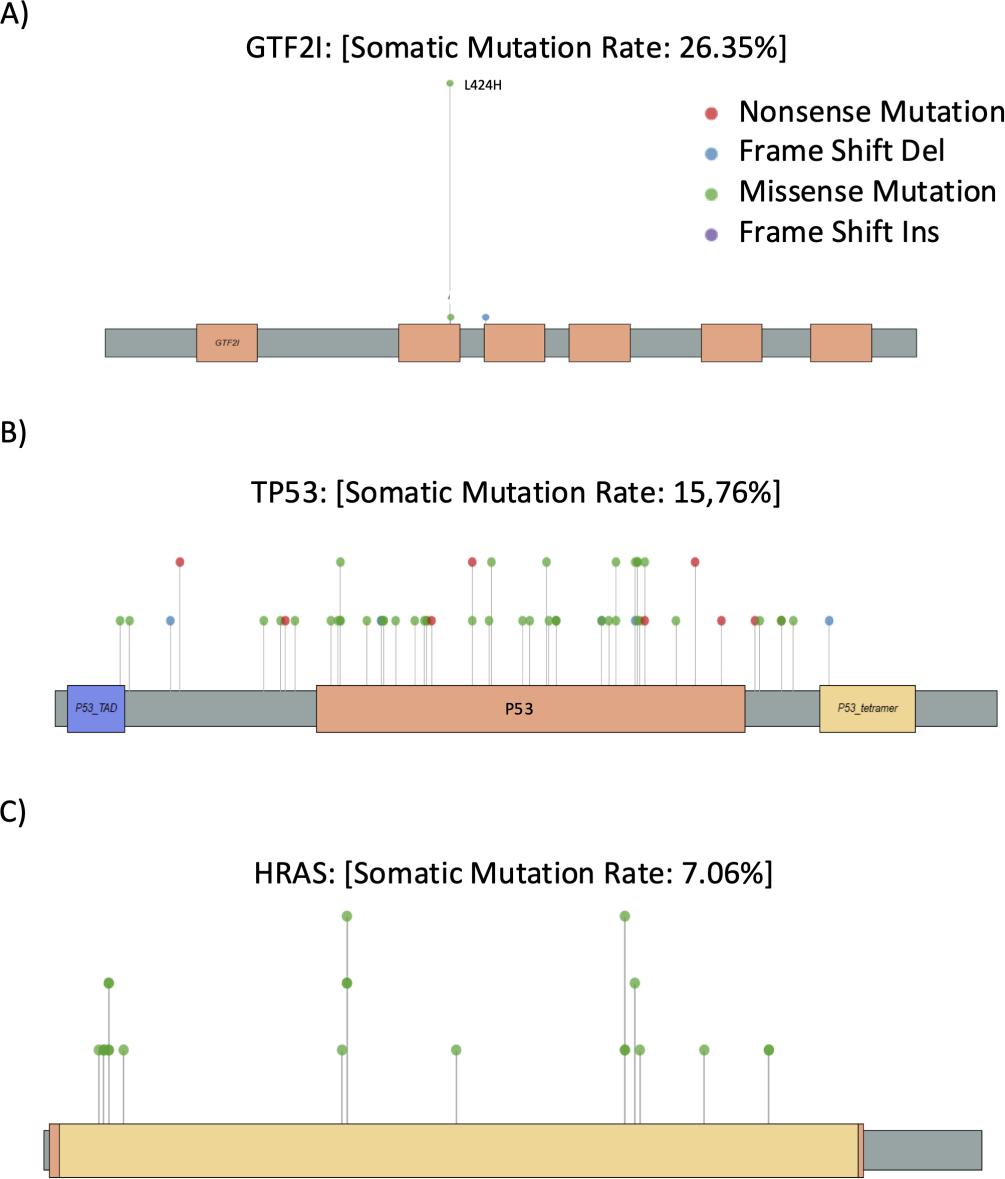
Lollipop Plots of most frequently mutated genes: Schematic representation of the linear distribution and frequency of somatic mutations across the protein structures of the 3 most mutated driver genes. Each “lollipop” represents an individual mutation, with the height of the stem indicating the mutation frequency at the corresponding amino acid residue. Mutation types are color-coded: green for missense, red for nonsense, and blue or purple for frameshift deletions and insertions. Colored bars along the horizontal axis indicate the known conserved functional domains of each protein.

We identified 566 INDELs and 4575 SNVs, of which 2945 were transitions (64.4%) and 1630 transversions (35.6%). Most of the mutations were missense 53.8%, followed by synonymous and stop gain mutations, whereas frameshift deletions were the most common INDELS (**Table 2**). Using the MAF tool, mutations in the coding sequence were predominantly missense, with C>T transitions outnumbering other mutation types (**Figures 2B, C**). **Figure 2E** reports the Oncoplot of the 20 most frequently mutated genes in TET. Indeed, GTF2I was the most mutated, with a unique missense substitution at L424H (**Figure 3A**). It is worth noting that the L424H mutation is related to the MANE transcript of GTF2I, which encodes the TFII-I alpha isoform. However, in TETs, we observed the expression of only the beta and delta TFII-I isoforms resulting from alternative splicing of exons 10 and 12. Therefore, the mutation corresponds to L404H in the beta isoform and L383H in the delta isoform. GTF2I mutations are prevalent in micronodular tumors and A and AB thymomas. TP53 was the second most frequently mutated gene, with mutations dispersed throughout the gene, consistent with its oncosuppressive function (**Figure 3B**). TP53 mutations were prevalent in thymic carcinomas. In the table of co-occurring and mutually exclusive mutations (**Figure 2D**), the presence of GTF2I and TP53 mutations was mutually exclusive, suggesting a TET population with distinct molecular aberrations. In tumors with GTF2I mutations, HRAS mutations frequently co-occurred. HRAS mutations are often non-canonical mutations of codon G12. Indeed, among 30 tumors with HRAS mutations, 23% had codon 13 mutations, 20% had codon Q61 mutations, and 17% had codon K117 mutations; only two mutations at codon G12 were observed (**Figure 3C** lollipop). On the other hand, APC, SMAD4, and SETD2 mutations co-occurred with TP53 mutations (**Figure 2D**).

**Table 2:** Effect of mutation on the coding sequence.

Exonic function	Mutation	Percentage
Nonsynonymous SNV	2765	53.8%
Synonymous SNV	954	18.6%
Stop gain	226	4.4%
Stoploss	5	0.1%
Start loss	4	0.1%
In-frame deletion	85	1.7%
In-frame insertion	29	0.6%
Non-frameshift substitution	2	0.0%
Frameshift deletion	218	4.2%
Frameshift insertion	50	1.0%
Frameshift substitution	1	0.0%
Noncoding	772	15.0%
Unknown	30	0.6%

SNV single-nucleotide variation.

### Estimation of mutation frequency

**Figure 4:**
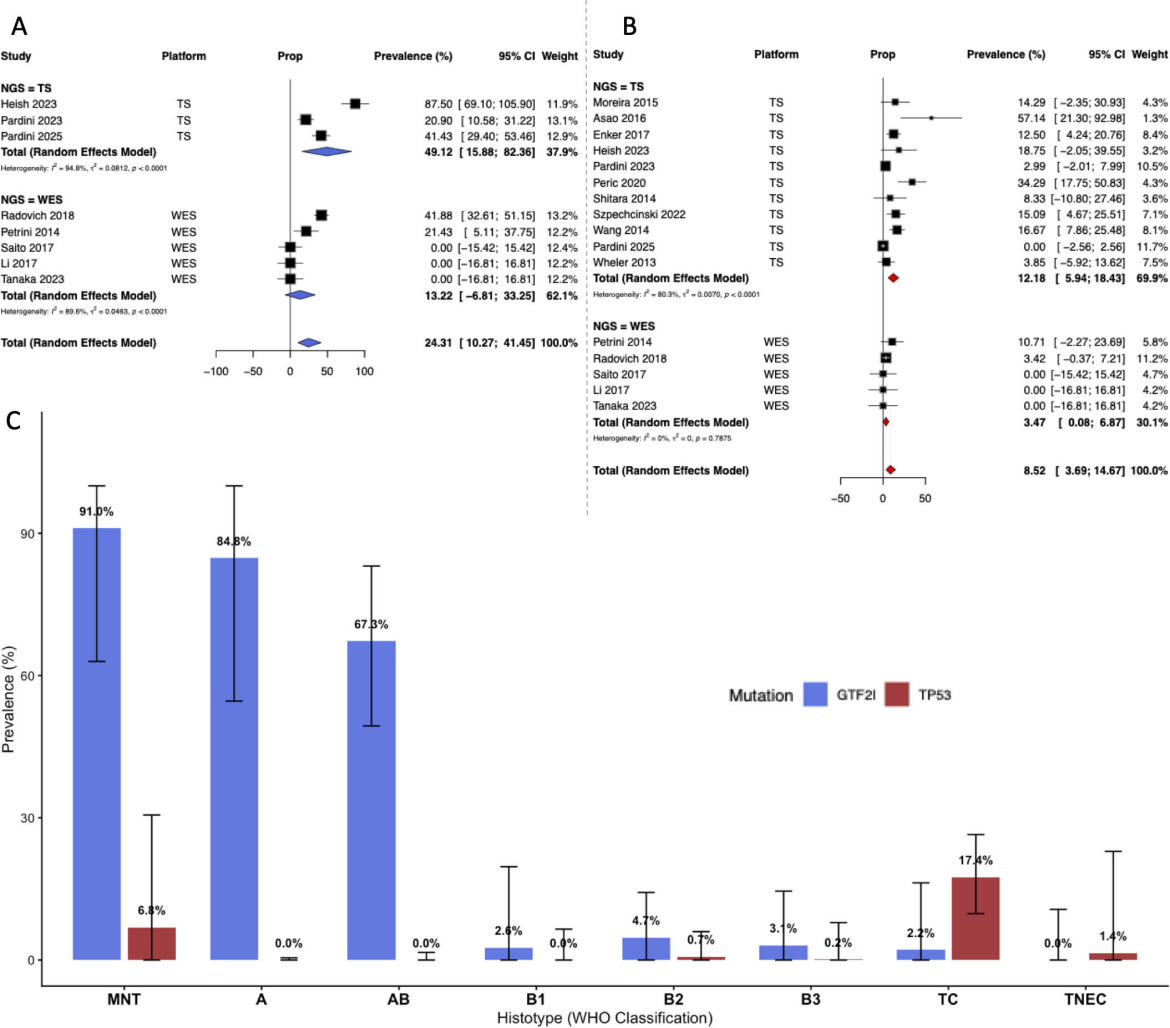
Estimation of the frequency of mutation of the most mutated genes in TET. Panel A presents a forest plot of the GTF2I mutation across studies that used whole-genome sequencing (WES) or targeted resequencing (TS). Using a random-effects model, the mutation frequency was 24.3%. Panel B presents a forest plot of the TP53 mutation across studies that used WES or TS. Using a random-effects model, the mutation frequency was 8.5%. Panel C depicts the frequency of GTF2I (blue) and TP53 (red) mutations in the most relevant histotypes of thymomas; error bars are the 95% confidence interval.

Significant heterogeneity in mutation frequency was observed across studies, particularly for GTF2I and TP53, likely reflecting their differing prevalence across histotypes (**Table 3**). GTF2I was the most frequently mutated gene, with a pooled prevalence of 24.31% (95% CI: 10.27%–41.45%). This gene also showed the highest heterogeneity (I^2^ = 87.9%, p < 0.0001), suggesting a preferential association with specific histological subtypes (**Figure 4A**). The meta-analysis of GTF2I mutation prevalence across different TET histotypes confirmed a significant pathological correlation (p < 0.0001). The highest mutation rates were observed in MNT (91.0%; 95% CI: 63.0%–100.0%) and Type A thymomas (84.8%; 95% CI: 54.6%–100.0%), followed by AB (67.3%; 95% CI: 49.4%–83.1%). In contrast, GTF2I mutations were rare or absent in more aggressive histotypes, with prevalence rates dropping significantly in Type B1 (2.6%), B2 (4.7%), B3 (3.1%), and Thymic Carcinoma (2.2%). Significant heterogeneity was observed only in A thymomas, supporting the idea that differences in histotype prevalence across studies are the major driver of the observed heterogeneity (**Supplementary Figure 1A**).

**Table 3:** Estimated frequency of the most mutated genes.

Gene	Studies included	Prevalence (Random Effect)	95% CI	Heterogeneity (I2)	p-value (Test Q)	P-value (Subgroup TS vs WES)
GTF2I	8	**24.3%**	10.3% - 41.6%	87.9%	**< 0.0001**	**0.0489**
TP53	16	**8.5%**	3.7% - 14.7%	76.6%	**< 0.0001**	**0.0194**
HRAS	14	**3.5%**	1.6% - 6.0%	16.8%	0.2702	0.0664
SETD2	9	**3.2%**	0.4% - 7.6%	59.4%	**0.0116**	0.1259
BCOR	8	**3.1%**	1.1% - 5.7%	0.0%	0.5416	0.0993
CYLD	10	**1.9%**	0.00% - 6.0%	61.7%	0.0052	0.1861
ATM	13	**1.3%**	0.0% - 3.6%	37.3%	0.0849	0.6717
TET2	10	**1.3%**	0.1% - 3.6%	21.6%	0.244	0.626
CDKN2A	13	**0.5%**	0.0% - 1.7%	0.0%	0.5194	0.8825
KIT	14	**0.4%**	0.0% - 1.6%	0.0%	0.7016	0.2932

We evaluated heterogeneity in mutation frequency across the studies. We included only studies evaluating a specific gene. For example, in the case of GTF2I, most targeted resequencing studies did not include this gene because it is specific to thymomas. Moreover, reports of only 1 patient were excluded because it was not estimable the frequency of mutation. The prevalence of mutation was calculated using a random effect model after a Freeman-Tukey double arcsine transformation. Heterogeneity was assessed using a Q test; a p-value <0.05 indicates significant heterogeneity among studies. According to Cochrane recommendations, I² values of 25%, 50%, and 75% were considered to represent low, moderate, and high heterogeneity, respectively, while values exceeding 75% indicated very high heterogeneity. We tested differences in heterogeneity between studies adopting targeted resequencing (TS) and whole exome sequencing (WES).

Bold numbers indicate the estimated mutation frequency according to the random-effect model, values showing significantly different heterogeneity for specific genes, and values demonstrating a significant difference between the employed sequencing technologies.

TP53 was the second most common alteration, with a pooled prevalence of 8.52% (95% CI: 3.69%–14.67%), and showed substantial heterogeneity (I^2^ = 76.6%), consistent with its recognized involvement in more aggressive phenotypes such as TCs (**Figure 4B**). The meta-analysis for TP53 mutations indicates a significant association with more aggressive histological subtypes (p = 0.0216). The highest pooled prevalence was observed in TCs at 17.45% (95% CI: 9.77%–26.45%), followed by NOS (9.30%) and MNT (6.85%). Notably, TP53 mutations were virtually absent in Type A (0.00%; 95% CI: 0.00%–0.47%) and Type AB thymomas (0.00%; 95% CI: 0.00%–1.60%). The B thymomas showed negligible mutation rates, with B1, B2, and B3 all remaining below 1% (**Figure 4C**; **Supplementary Figure 1B**).

SETD2 and HRAS followed with pooled prevalences of 3.16% and 3.50%, respectively. Notably, BCOR mutations (3.06%) showed remarkable consistency across cohorts (I^2^ = 0.0%, p = 0.54), as did HRAS mutations (I^2^ = 16.8%, p = 0.27). Other genes involved in DNA damage response and tumor suppression, including CYLD (1.92%), ATM (1.31%), and TET2 (1.29%), were identified as relatively uncommon alterations. Classic oncogenic drivers such as KIT and CDKN2A were extremely rare, with pooled prevalence of 0.44% and 0.47%, respectively, and no heterogeneity (I^2^ = 0.0%). Subgroup analysis based on sequencing technology (targeted sequencing vs. whole exome sequencing) revealed significant differences for GTF2I, with targeted sequencing showing higher mutation detection rates than whole exome sequencing (48.17% vs. 10.03%; p = 0.0489), and even more pronounced differences for TP53 (11.94% vs. 1.90%; p = 0.0194). Significant technological discrepancies were also observed for SETD2 (p = 0.0059 for the common effect) and CYLD (p = 0.0052), with targeted sequencing generally yielding higher frequencies. Conversely, no statistically significant differences were observed among sequencing approaches for HRAS, KIT, and BCOR (p > 0.05), suggesting that sequencing methodology did not substantially affect mutation detection for these markers.

### Pathway analysis

**Figure 5:**
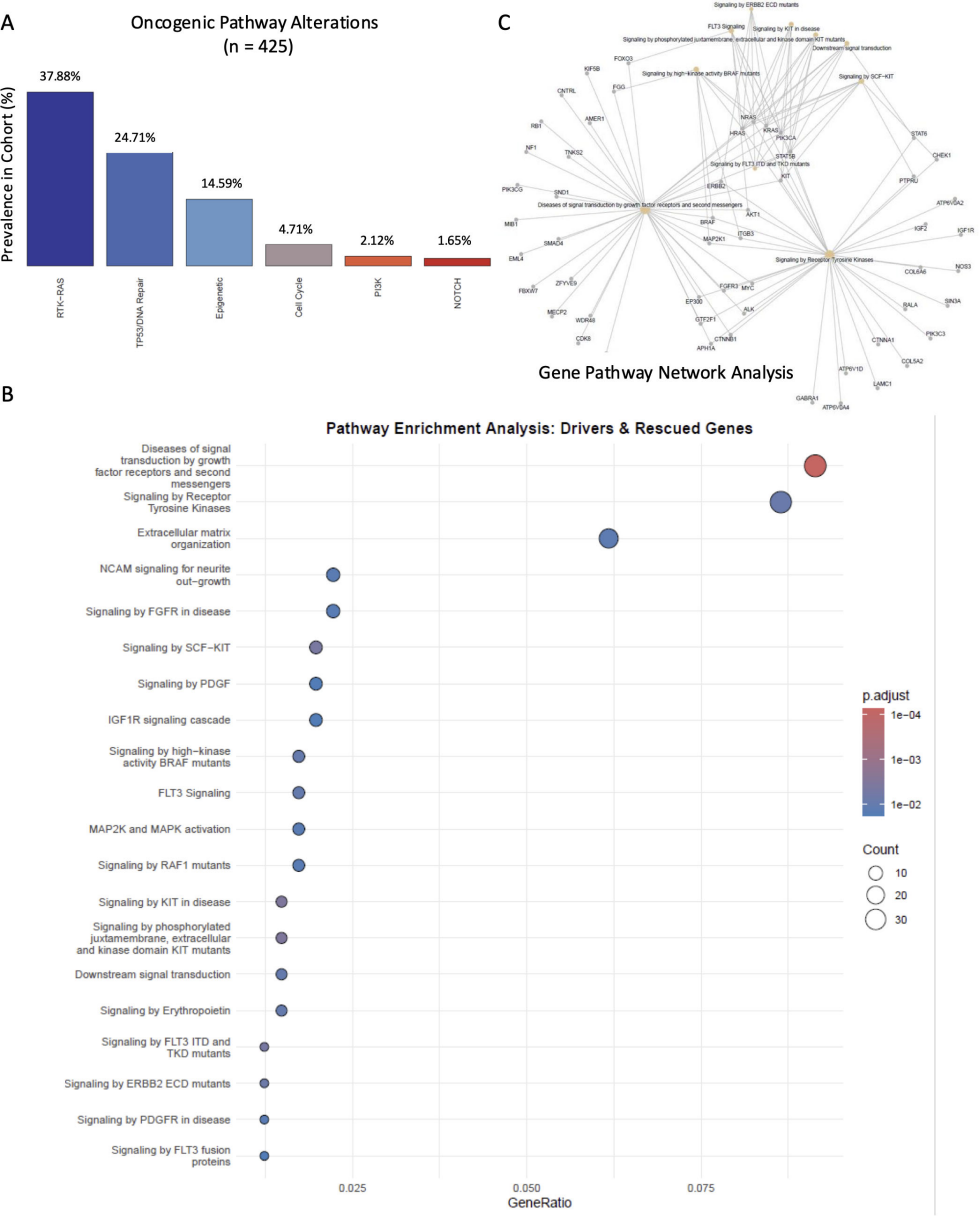
Characterization of genomic alterations and oncogenic signaling pathways. **(A)** Bar chart showing the prevalence of alterations across major oncogenic pathways in the cohort. The RTK–RAS pathway was most frequently affected (37.88%), followed by TP53/DNA repair (24.71%) and epigenetic regulators (14.59%). Alterations in the cell cycle, PI3K, and NOTCH pathways occurred at lower frequencies. **(B)** Dot plot of Reactome analysis showing the gene ratio on the x-axis, dot color indicating statistical significance, and dot size representing the number of genes in each pathway. Enrichment is observed primarily in signal transduction and RTK signaling pathways. **(C)** Gene-Concept Network (Cnetplot) diagram highlighting interactions between mutated genes and their associated pathways. Hub nodes identify key genes and processes integrating growth factor signaling and transcriptional regulation.

The oncogenic pathway analysis using the dataset without synonymous mutations demonstrated enrichment of the RTK/RAS pathway (37.9% of TETs), TP53 & DNA repair (24.7%), and epigenetic regulators (14.6%) (**Figure 5A**).

The integration of statistical clustering and manual curation revealed a robust mutational architecture. OncodriveCLUST identified 10 primary drivers, with HRAS showing the highest clustering score (0.92, FDR = 6.31 x10^-6), followed by NRAS and KRAS. The inclusion of curated genes confirmed GTF2I as the most frequent alteration and highlighted a significant burden among epigenetic regulators, including SETD2 and KMT2D.

The Reactome analysis provided a detailed map of the altered molecular pathways (**Figure 5B**). The most significant enrichment was found in “Diseases of signal transduction by growth factor receptors” (adjusted p-value= 7.4 x 10^-5), encompassing signaling hubs for KIT, FGFR, and ERBB2 (**Supplementary Table 4**). Chromatin remodelers revealed a significant enrichment in “Chromatin organization” (adjusted p-value = 0.042) and “Formation of WDR5-containing histone-modifying complexes” (adjusted p-value = 0.039). This indicates that the mutational landscape of our cohort is defined by two central functional poles: Signal Transduction Hubs: Dominated by the RAS family and RTK mutants (KIT/FGFR3), driving mitogenic cascades (**Supplementary Table 4**). Epigenetic Reprogramming: Centered on SETD2, ASXL1, and KMT2D, likely affecting global gene expression patterns. From the Cnetplot analysis (**Figure 5C**), we observed a robust mutational convergence on pathways critical for cell survival and proliferation, revealing a highly interconnected molecular architecture. The network is primarily dominated by the hubs centered on Receptor Tyrosine Kinase signaling and growth factor-mediated signal transduction. Master connectors such as HRAS, KRAS, NRAS, and PIK3CA act as universal bridges, linking membrane receptors to multiple downstream effectors, including AKT1 and BRAF. Furthermore, a significant portion of the network is dedicated to specific oncogenic axes involving KIT (including SCF-KIT and mutant signaling) and ERBB2 (notably ECD mutants), suggesting these receptors represent key vulnerabilities that converge on the identical intracellular transducers. The analysis also demonstrates functional integration between cytoplasmic signal transduction and nuclear gene regulation, with nodes such as EP300, MYC, and CTNNB1 serving as interfaces for potential transcriptional reprogramming. Finally, the network identifies involvement in diverse regulatory processes, including cell cycle control (RB1, CHEK1) and JAK/STAT signaling (STAT5B, STAT6), highlighting the pleiotropic effects of the somatic mutations identified in our cohort.

According to our table of co-occurring mutations, we divided TETs into tumors with GTF2I mutations (110), tumors with TP53 mutations (63), and double-negative tumors (DN) without GTF2I or TP53 mutations (247). Thereafter, we conducted Reactome pathway analysis across the three distinct TET groups and compared the results (**Supplementary Tables 5-8**, **Supplementary Figure 2**). Our enrichment analysis revealed three biological profiles that correlated with the mutational status. The GTF2I-mutated group, typically associated with indolent clinical behavior, exhibited fewer mutations than the other subgroups, highlighting GTF2I as a driver of tumor growth. A particular enrichment in pathways related to specialized cell-cell adhesion was observed: the most significant pathways included Protein-protein interactions at synapses and NCAM signaling for neurite out-growth (adjusted p-value = 0.015). Mutations in genes including NRXN1, NLGN4X, PTPRD, and GRIN2A drove this profile (**Supplementary Table 5**).

The TP53-mutated tumors exhibited more mutations, providing a signature of high aggressiveness and proliferative signaling. Enrichment was observed in Diseases of signal transduction by growth factor receptors and Signaling by Receptor Tyrosine Kinases (adjusted p-value < 0.0001). Oncogenic drivers of this pathway include FGFR (1–3), ERBB2, ALK, EGFR, KIT, and PDGFR. These receptors converged on the RAF/MAPK and PI3K/AKT cascades, facilitating rapid cell cycle progression and survival (**Supplementary Table 6**).

The DN group, lacking GTF2I or TP53 mutations, demonstrated a profile centered on the tumor microenvironment and metabolic reprogramming. A dominant signature was found in extracellular matrix Organization and Collagen chain trimerization (adjusted p-value = 0.007). Moreover, a significant enrichment was observed in Epigenetic regulation of gene expression (notably the WDR5-containing histone-modifying complexes) and Adipogenesis/PPAR-alpha signaling (**Supplementary Table 7**).

To ensure the robustness of our functional characterization, enrichment analysis was cross-validated using both Reactome and KEGG databases. This dual approach enabled comprehensive mapping of both detailed biochemical reactions (Reactome) and systemic biological pathways (KEGG). The high degree of concordance between the two databases, specifically regarding the neuro-mimetic adhesion signature in the GTF2I cluster and the oncogenic, multi-kinase signaling (notably the PI3K-Akt/Ras axis) in the TP53 cluster, strengthens the biological validity of our molecular stratification and highlights distinct therapeutic vulnerabilities for each subgroup (**Supplementary Tables 9-11**, **Supplementary Figure 3**).

### Mutational signature analysis

**Figure 6:**
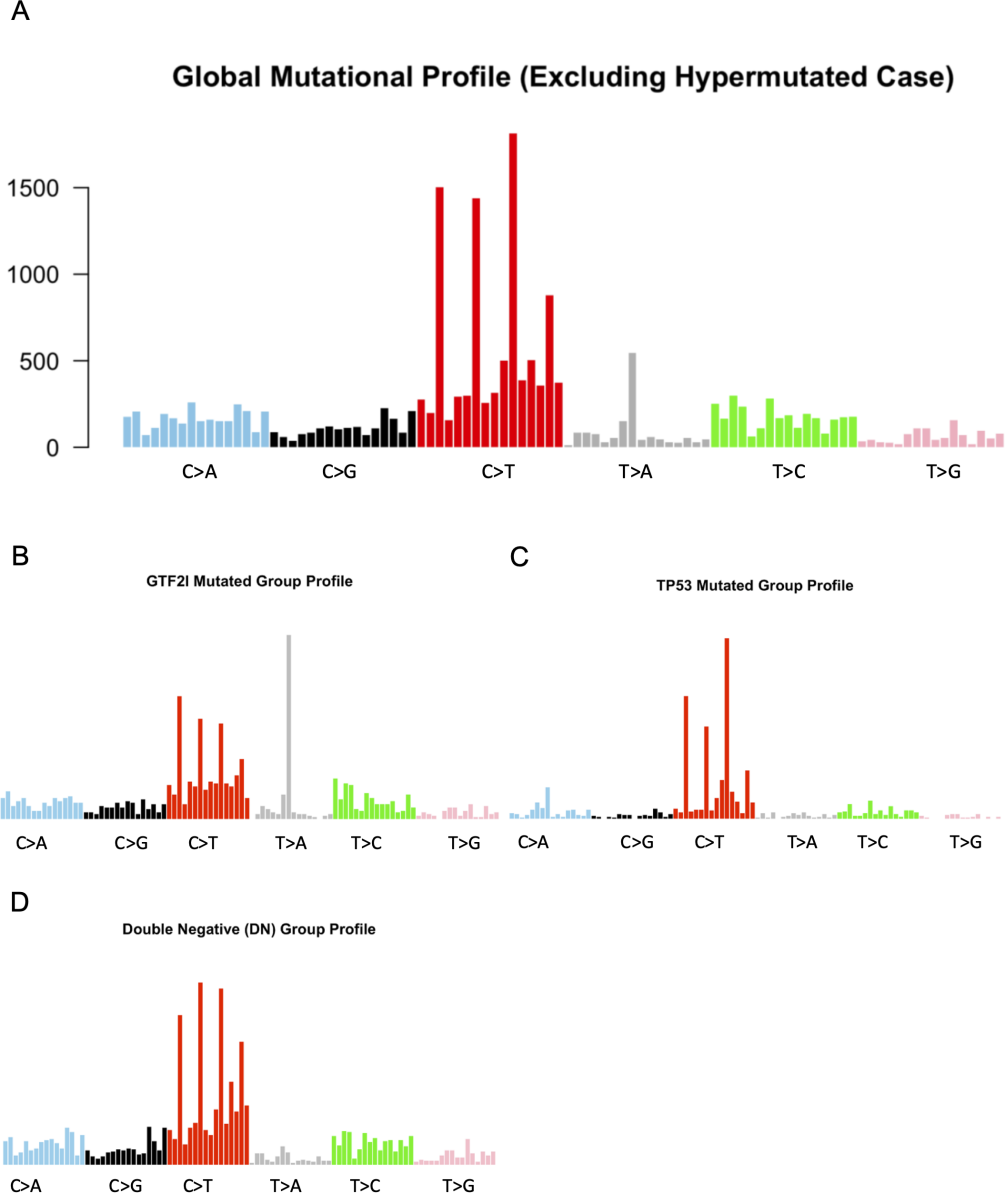
Mutational signature profiles across subgroups. **(A)** Global mutational profile: Distribution of single-base substitutions across the entire cohort (excluding hypermutated cases), showing a predominance of C>T transitions. **(B)** GTF2I-mutated subgroup: Mutational spectrum specific to GTF2I-mutated cases, characterized by distinct peaks in C>T and T>A substitutions. **(C)** TP53-mutated subgroup: Signature of TP53-mutated cases. **(D)** Double-negative (DN) subgroup: Mutational profile of samples lacking both GTF2I and TP53 mutations.

To investigate the etiological mutational processes in TETs, we performed a mutational signature analysis of somatic substitutions using a signature-fitting approach against the COSMIC v2 database. We excluded a single tumor from this analysis, with 936 mutations due to mismatch repair deficiency, as reported in the TCGA article (16). The analysis revealed a mutational landscape predominantly driven by endogenous biological processes (**Figure 6A**; **Supplementary Table 12**). Signature 1, associated with the spontaneous deamination of 5-methylcytosine and correlated with chronological age, emerged as the dominant driver with a perfect cosine similarity score (1.00). High similarity scores were also observed for Signature 2 (0.919), typically associated with APOBEC enzymatic activity, and for Signature 7 (0.882), suggesting a complex interplay of endogenous mutagenic pressures. Notably, the contribution of Signature 6 (0.62) and Signature 15 (0.648), both linked to DNA mismatch repair, was detectable but secondary to the clock-like aging profile. These findings indicate that genomic instability in this TET cohort arises primarily from cumulative endogenous DNA damage and age-related mutational drift, rather than from exposure to exogenous carcinogens or from specific DNA double-strand break repair deficiencies, as previously hypothesized.

Thereafter, we repeated the mutational signature-fitting approach across the GTF2I-mutated, TP53-mutated, and DN groups (**Figure 6B–D**; **Supplementary Table 13**). After excluding the hypermutated MMR-deficient case to avoid bias, the mutational landscape continued to show distinct group-specific patterns. The GTF2I-mutated and DN groups were primarily characterized by Signature 1 (spontaneous deamination of 5-methylcytosine), reflecting a clock-like endogenous process correlated with age (cosine similarity 0.735 and 0.892, respectively). In striking contrast, the TP53-mutated subgroup exhibited a highly distinct and robust profile. It was defined by a predominant enrichment of Signature 6, conventionally associated with defective DNA mismatch repair (MMR), with an exceptionally high cosine similarity of 0.923. This finding suggests that in TETs, TP53 mutations define a specific mutational phenotype characterized by profound DNA repair instability, which persists as a group-wide feature even after accounting for the hypermutated outlier.

## Discussion

A large mutational dataset of TETs has been assembled for this meta-analytic approach, enabling the identification of distinct molecular subgroups that distinguish biologically and clinically meaningful heterogeneity within TETs.

Consistent with prior studies, GTF2I emerged as the most frequently mutated gene in thymomas, particularly in micronodular (91%), type A (84.8%), and type AB (67.3%) tumors, which are usually diagnosed at an early stage. The overall mutation frequency of GTF2I in our cohort was consistent with previous reports, 82% in type A and 74% in AB thymomas. Mutated GTF2I is an oncogene in thymic epithelial tumors. Indeed, transgenic mice that express GTF2I with L424H mutation selectively in thymic epithelial cells under the FOXN1 promoter develop thymomas (32, 33). Our findings further support GTF2I as a key oncogenic driver in thymomas, characterized by a low tumor mutational burden and a distinctive pathway profile. Notably, although GTF2I mutations are typically associated with indolent disease, we also identified a subset of advanced-stage (IV-B) tumors harboring GTF2I mutations and a higher mutational burden, suggesting a potential need for targeted therapies for these tumors.

The recurrent co-occurrence of HRAS mutations within this group may reflect a multistep model of thymoma tumorigenesis. Network-based analyses revealed that HRAS functions as a molecular bridge between NCAM-mediated adhesion and cell-survival signaling, whereas structural proteins such as SPTAN1 and NRXN1 emerged as central hubs. From a clinical perspective, while these tumors may theoretically be sensitive to MEK inhibition, their slow growth kinetics and high degree of structural organization support surgical resection as the primary therapeutic strategy, with molecularly targeted therapies reserved for less common cases that result in unresectable or progressive disease.

In contrast, TP53-mutated tumors predominantly comprised TCs (17.4%) and showed a distinct co-mutation pattern involving APC, SMAD4, and SETD2. These tumors exhibited a higher tumor mutational burden and a more aggressive clinical phenotype. Indeed, TP53 mutations are more common in TCs, and tumors are frequently diagnosed at advanced stages (16). Network analysis of this cluster revealed a high degree of signaling convergence, with GRB2 and PIK3R1 serving as critical intracellular bottlenecks that integrate signals from multiple overactive receptor tyrosine kinases, including FGFR1, ALK, and ERBB2. This convergence onto the MAPK/ERK axis provides a mechanistic explanation for the aggressive behavior of these tumors and suggests that targeting a single upstream receptor may be insufficient. Instead, the extensive pathway redundancy observed in this subgroup supports the rationale for downstream or combinatorial therapeutic strategies, such as pan-kinase inhibition or combined PI3K/mTOR and MEK blockade, to overcome signaling plasticity in TP53-mutated TETs.

Tumors lacking mutations in both GTF2I and TP53 represented a molecularly heterogeneous group with intermediate characteristics. The interpretation of this cluster is complicated by intrinsic features of TETs, including low tumor mutational burden and the frequent presence of abundant non-neoplastic thymocytes, which can dilute tumor DNA and hinder the detection of somatic mutations. Hub-based network analysis highlighted a dual dependence on WDR5, a key epigenetic regulator, and on basement membrane components, such as COL4A1. The prominence of PPARG further suggests a metabolic shift toward lipid regulation, distinguishing this subgroup from TP53-mutated tumors driven by kinases. These findings point to potential epigenetic vulnerabilities in double-negative tumors and suggest that therapies targeting chromatin remodeling or the tumor microenvironment, including extracellular matrix organization and angiogenesis, may be more effective in this subgroup.

Interestingly, mutational signatures correlated with the proposed molecular stratification. GTF2I-mutated and double-negative tumors were enriched for age-related mutational processes, whereas TP53-mutated tumors exhibited signatures associated with DNA repair defects, including mismatch repair–related patterns. Although one hypermutated TP53-mutant case strongly influenced the MMR signal, the overall trend aligns with the higher mutational burden and aggressive clinical behavior observed in this group. These findings are consistent with epidemiological observations: thymomas (particularly type A and AB) typically arise in the sixth to seventh decade of life, whereas thymic carcinomas often present at a younger median age.

Differences in oncogenic pathways that sustain tumor growth across molecular subgroups could have clinical implications. Thymic carcinomas, which are more frequently mutated, have benefited from advances in targeted therapies developed for other solid tumors in recent clinical trials (34–37). In contrast, thymomas, frequently GTF2I-mutated or DN, have seen fewer therapeutic breakthroughs in the last two decades. Molecular characterization of GTF2I and TP53 should be incorporated into future clinical trials of TETs to assess differential therapeutic responses and to validate the clinical relevance of this molecular classification. Furthermore, pathway-level analyses suggest novel, subgroup-specific therapeutic strategies that warrant prospective investigation.

This study has several inherent limitations. The heterogeneity of sequencing technologies introduces interpretative challenges, as our dataset includes whole-exome (2, 13–18) and whole-genome sequencing (19, 20) approaches, thymoma-focused targeted panels (21, 22), and generic cancer panels with variable gene counts (23–31). Notably, some panels did not include GTF2I. Another limitation is the unavailability of large, extended-panel datasets (3, 38), which could not be incorporated because they lack the variant-level data required for this analysis. Our analysis focuses on somatic mutations and excludes copy number aberrations. However, deletion of the CDKN2A locus significantly contributes to the inactivation of this critical tumor suppressor gene and is underrepresented in our analysis. Moreover, the deletion of the CDKNA locus could be a target for MTAP inhibitors. Finally, our other view is hypothesis-generating, since confirmatory validation in an independent cohort and in biological models has not yet been provided within this article.

In conclusion, this large-scale integrative analysis provides a refined view of the mutational landscape of thymic epithelial tumors, supporting their classification into three molecularly distinct groups with potential clinical relevance. This framework may inform future biological studies and guide the development of more effective, molecularly tailored therapeutic strategies for TETs.

## Data Availability

The original contributions presented in the study are included in the article/**Supplementary Material**. Further inquiries can be directed to the corresponding author.
