# Supplementary material for: Genomic landscape and subgroup stratification of thymic epithelial tumors: a systematic meta-analysis of next-generation sequencing data
Source: Front Oncol. 2026 Feb 26;16:1781510. doi: 10.3389/fonc.2026.1781510 (PMC12979072; doi:10.3389/fonc.2026.1781510)
Supplement: Supplementary file 1 [file DataSheet1.docx]

**Supplementary Figures and Tables.**

**Supplementary Table S1.** Studies included in the meta-analysis

**Supplementary Table S2.** Patients’ characteristics

**Supplementary Table S3.** Unique mutations

**Supplementary Table S4.** Significant pathways in Reactome analysis

**Supplementary Table S5.** Significant pathways in Reactome analysis of Double Negative TETs

**Supplementary Table S6.** Significant pathways in Reactome analysis of GTF2I mutated TETs

**Supplementary Table S7.** Significant pathways in Reactome analysis of TP53 mutated TETs

**Supplementary Table S8.** Comparison of Reactome Pathway Statistics

**Supplementary Table S9.**  KEGG Results of GTF2I mutated TETs

**Supplementary Table S10.**  KEGG Results of TP53 mutated TETs

**Supplementary Table S11.**  KEGG Results of Double Negative mutated TETs

**Supplementary Table S12.** Signature Results of TETs

**Supplementary Table S13.** Signature Comparison: GTF2I vs TP53 vs Double Negative

**Supplementary Figure 1.**

**Supplementary Figure 1:** reports the estimation of the frequency of mutation of GTF2I (A) AND TP53 (B) according to TET histotypes in the evaluated studies.

**Supplementary Figure 2.**

**
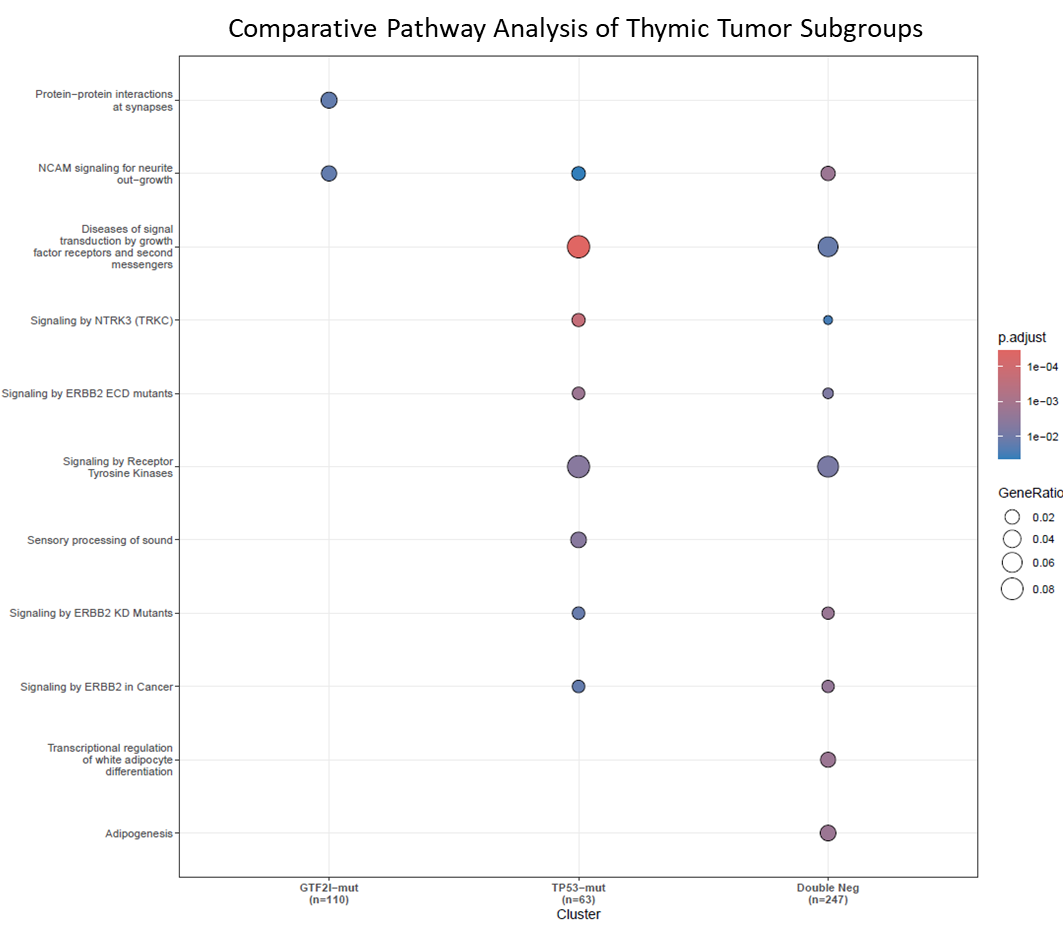
**

**Supplementary Figure 2.** Dot plot summarizing pathway enrichment analysis across three thymic tumor molecular subgroups: GTF2I mutated, TP53 mutated, Double Negative. Pathways are shown on the y-axis and tumor clusters on the x-axis.

**Supplementary Figure 3.**

**
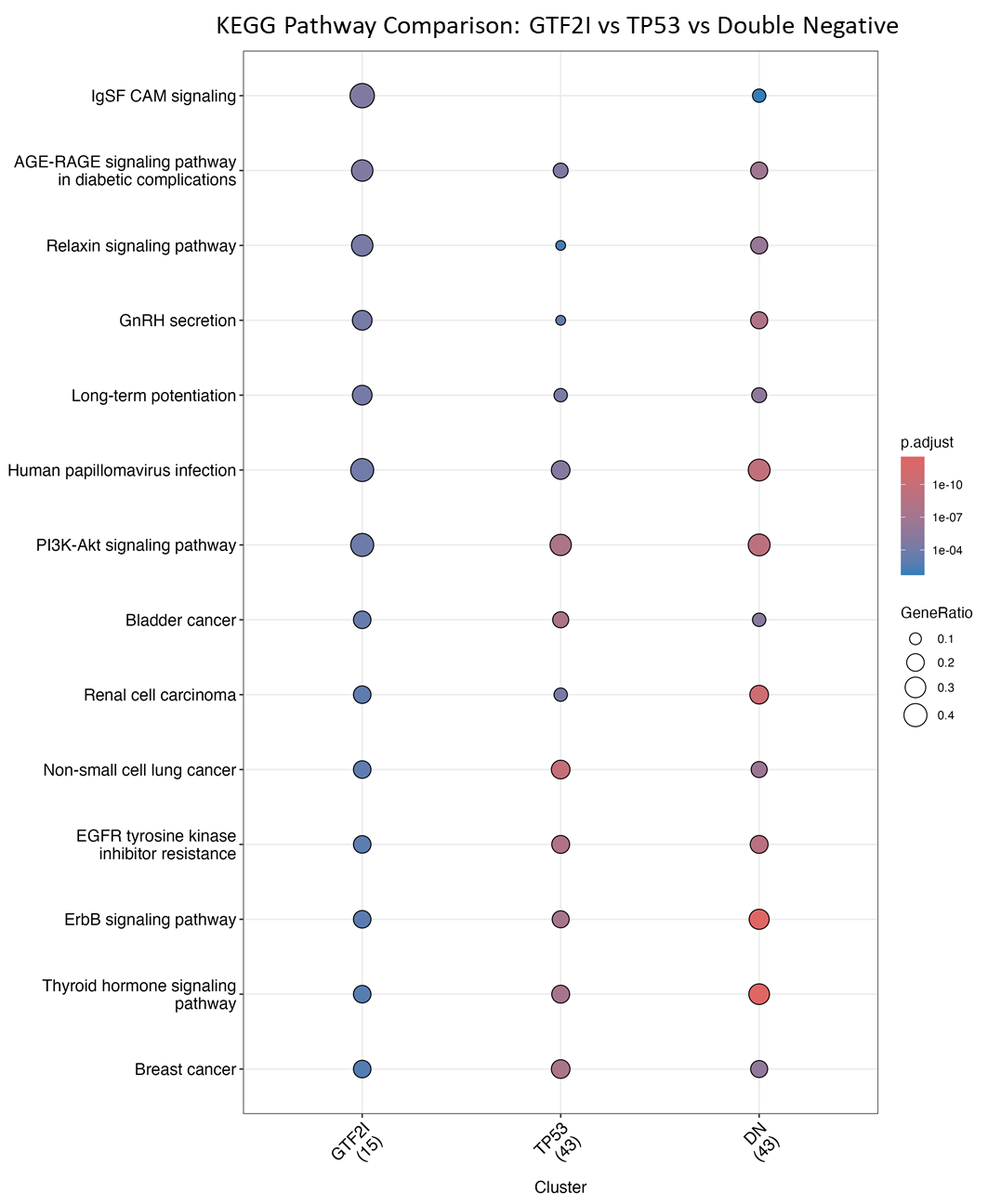
**

**Supplementary Figure 3.** Dot plot showing KEGG pathway enrichment across thymic tumor molecular clusters (GTF2I-mutant, TP53-mutant, and double-negative). Pathways are displayed on the y-axis and clusters on the x-axis.
